# Using biodiversity databases to assess vascular plant diversity in Protected Areas: a case study of Parque Estadual do Forno Grande, Brazil

**DOI:** 10.3897/BDJ.12.e133976

**Published:** 2024-10-30

**Authors:** Thuane Bochorny, Guilherme M. Antar, Igor H.F. Azevedo, Fernando Bianchi Junior, Tatiana T. Carrijo, Valquiria F. Dutra, André P. Fontana, Claudio N. Fraga, Samuele Gerace, Leandro L. Giacomin, André S.B. Gil, Renato Goldenberg, Diego R. Gonzaga, Gustavo Heiden, Ingrid Koch, Ludovic J.C. Kollmann, Paulo H. Labiak, Duane F. Lima, Gabriel M. Marcusso, Pedro L.R. Moraes, Filipe Torres-Leite, Pedro L. Viana, Rafaela C. Forzza

**Affiliations:** 1 Jardim Botânico do Rio de Janeiro, Rio de Janeiro, Brazil Jardim Botânico do Rio de Janeiro Rio de Janeiro Brazil; 2 Centro Universitário Norte do Espírito Santo, São Mateus, Brazil Centro Universitário Norte do Espírito Santo São Mateus Brazil; 3 Universidade Federal de Mato Grosso, Cuiabá, Brazil Universidade Federal de Mato Grosso Cuiabá Brazil; 4 Universidade Estadual Feira de Santana, Feira de Santana, Brazil Universidade Estadual Feira de Santana Feira de Santana Brazil; 5 Universidade Federal do Espírito Santo, Alegre, Brazil Universidade Federal do Espírito Santo Alegre Brazil; 6 Universidade Federal do Espírito Santo, Vitória, Brazil Universidade Federal do Espírito Santo Vitória Brazil; 7 Operações Paisagísticas, Santa Teresa, Brazil Operações Paisagísticas Santa Teresa Brazil; 8 Universidade Federal da Paraíba, João Pessoa, Brazil Universidade Federal da Paraíba João Pessoa Brazil; 9 Museu Paraense Emilio Goeldi, Belém, Brazil Museu Paraense Emilio Goeldi Belém Brazil; 10 Universidade Federal do Paraná, Curitiba, Brazil Universidade Federal do Paraná Curitiba Brazil; 11 Universidade Federal do Oeste do Pará, Santarém, Brazil Universidade Federal do Oeste do Pará Santarém Brazil; 12 Embrapa Clima Temperado, Pelotas, Brazil Embrapa Clima Temperado Pelotas Brazil; 13 Universidade Estadual de Campinas, Campinas, Brazil Universidade Estadual de Campinas Campinas Brazil; 14 Instituto Nacional da Mata Atlântica, Santa Teresa, Brazil Instituto Nacional da Mata Atlântica Santa Teresa Brazil; 15 Universidade Federal de Santa Catarina, Florianópolis, Brazil Universidade Federal de Santa Catarina Florianópolis Brazil; 16 Universidade Estadual Paulista, Rio Claro, Brazil Universidade Estadual Paulista Rio Claro Brazil; 17 Instituto Chico Mendes de Conservação da Biodiversidade, Prado, Brazil Instituto Chico Mendes de Conservação da Biodiversidade Prado Brazil

**Keywords:** Atlantic Forest, conservation, Catálogo de Plantas das Unidades de Conservação do Brasil, Protected Areas, taxonomy

## Abstract

**Background:**

The Parque Estadual do Forno Grande is a fully protected area in the southern Espírito Santo State, Brazil. It belongs to the Atlantic Forest domain, with predominantly dense, ombrophilous, seasonal semi-deciduous forests and herbaceous/shrubby vegetation on rock outcrops. The area is recognised as highly important for conservation, designated as a priority biological area for protecting the Atlantic Forest's biodiversity. Although the importance of Protected Areas in conserving the Atlantic Forest biodiversity is unquestionable, it is crucial to understand the floristic patterns within these regions to develop effective conservation strategies. We utilised national online databases to compile species lists containing relevant information about their biodiversity. The updated list of vascular plants recorded in the Parque Estadual do Forno Grande is available in the “Catálogo de Plantas das Unidades de Conservação do Brasil” and it is presented here with further information on richness, endemism and conservation status.

**New information:**

The Parque Estadual do Forno Grande harbours 958 species of vascular plants, of which 79.2% are angiosperms, 18.4% are ferns and 2.4% are lycophytes. Amongst these species, 44% are endemic to the Atlantic Forest. There are 58 threatened species, of which six are Critically Endangered, 39 are Endangered and 13 are Vulnerable. Amongst the threatened species, 51 are endemic to the Atlantic Forest. The number of records and the species richness in this area are notably high for Atlantic Forest standards. Our findings suggest that floristic inventories of Brazilian Protected Areas are a key contribution to the general perception of how much we still do not know about our flora. It also highlights the necessity of supporting floristic surveys in poorly-known areas, especially those remaining as forest remnants.

## Introduction

The Atlantic Forest has endured centuries of extensive habitat loss and fragmentation, leading to a dramatic reduction of native vegetation cover (Ribeiro et al. 2009). Today, it occupies only about 28% of its original area, dispersed across small, isolated forest fragments. Most of these remnants are covered with secondary vegetation, with less than half lying within protected areas (Rezende et al. 2018). The State of Espírito Santo is fully inserted in the Atlantic Forest domain and it is known for its diverse environments and landscapes. This domain encompasses various vegetation types including dense, ombrophilous and seasonal semi-deciduous forests, high-altitude grassland, savannah-steppe, *restinga* and mangrove (IBGE 2012, Garbin et al. 2017). In addition, the inselbergs in Espírito Santo are noteworthy for their unique flora, which significantly enhances the State's diversity and endemism (isolated monoliths of granitic and/or gneissic rock rising abruptly from the surrounding landscape - Barthlott and Porembski 2000, de Paula et al. 2020).

The historical degradation of native ecosystems in the sState and the limited understanding of biodiversity in many of the protected areas show us the urgency of investments in field expeditions to deepen the floristic knowledge within these protected regions (Oliveira et al. 2017; Dutra et al. 2022). An important contribution to the knowledge of the flora of Espírito Santo came from the studies of Dutra et al. 2015, Carrijo and Mansano (2017) and Dutra et al. (2022) which where based on data from virtual herbaria and the Flora and Funga of Brasil website (http://floradobrasil.jbrj.gov.br). The efforts constitute a reference for that State’s flora in terms of its floristic-taxonomic treatment, with morphological descriptions of 15% of the families, 11% of the genera and 12% of the species of angiosperms mentioned for Espírito Santo.

The Parque Estadual do Forno Grande (henceforth PEFG) is a protected area in the Municipality of Castelo, in the southern part of Espírito Santo. The park's vegetation consists of dense montane and high-montane ombrophilous forests, seasonal semi-deciduous forests and herbaceous/shrubby vegetation on rock outcrops by the inselbergs. The PEFG is of extreme biological importance, falling within the priority areas for conserving the Atlantic Forest's biodiversity (Conservation International do Brasil 2000, Fraga et al. 2019). The park is one of the few areas in Espírito Santo visited by naturalists in the 19^th^ and early 20^th^ centuries and, therefore, is one of the best-known in the State. For instance, the German-Brazilian botanist Alexander Curt Brade did extensive fieldwork in the region, discovering and describing several new species to science (whose collections are deposited in the RB herbarium). Since Brade’s first studies, many other botanists have done sporadic field trips to the region, which also resulted in the discovery of new species. Some examples include *Aciantherafornograndensis* L.Kollmann & A.P.Fontana, *Callianthecapixabae* M.T.R.Costa & Bovini, *Ctenitisglandulosa* E.S.Viveiros & Salino, *Mandevillafornograndensis* J.F.Morales, L.Kollmann & Fraga, *Megalastrumsubstrigosum* R.C.Moran et al., *Pabstiellarupicola* L.Kollman, *Selaginellamucronata* G.Heringer et al., *Pleromacastellense* (Brade) P.J.F.Guim. & Michelang., *Pleromafornograndense* F.S.Mey. et al., *Pleromatedescoi* (Meirelles et al.) P.J.F.Guim. & Michelang and *Rudgeaaxilliflora* Bruniera & Torres-Leite. Recent floristic studies conducted at the PEFG are rare, with the most notable being the Flora of Melastomataceae by Meirelles and Goldenberg (2012).

Although national online biodiversity databases (e.g. SiBBr, Reflora, Jabot, CNCFlora and SpeciesLink) have advanced in recent years, they tend to focus on particular goals, creating challenges in consolidating information on the flora of protected areas (Moreira 2020a). The Catalogue of Plants of Protected Areas in Brazil ("Catálogo de Plantas das Unidades de Conservação do Brasil”; https://catalogo-ucs-brasil.jbrj.gov.br/) was introduced in 2018 as an innovative tool designed to address the critical need for knowledge and access to information regarding the biodiversity within Brazil's Protected Areas (Moreira 2020a). It allows users to access herbarium images for most species, helping botanists in the identification process. It also provides access to the conservation status of each species since it is linked to the Brazilian National Center for Plant Conservation (CNCFlora, acronym in Portuguese for “Centro Nacional de Conservação da Flora”).

In this study, we provide and discuss information on the richness, endemism and conservation status of recorded species in Parque Estadual do Forno Grande - a rich and important area of endemism in the Brazilian Atlantic Forest - that was not presented in the Catalogue of Plants of Protected Areas in Brazil due to its limited scope.

## Sampling methods

### Study extent

**Figure 1.**
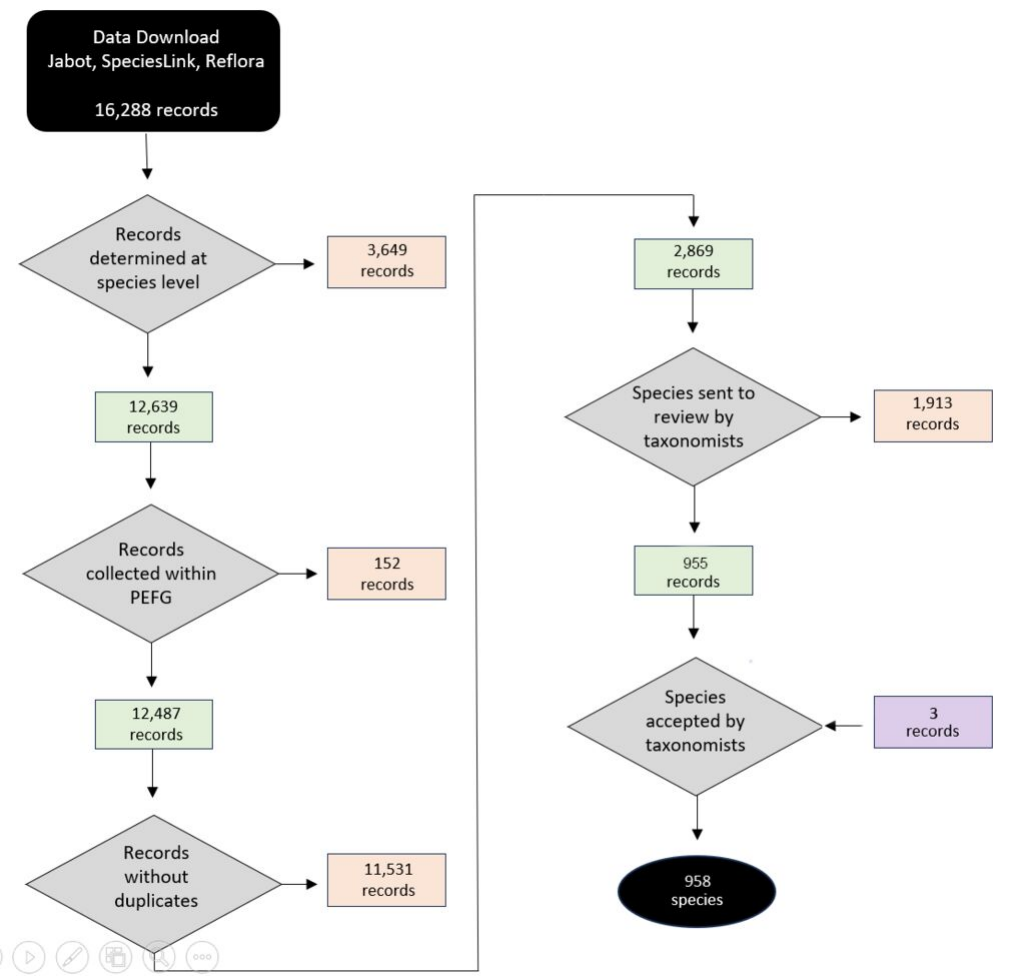
Workflow for data cleaning and elaboration of the species list for Parque Estadual do Forno Grande, Brazil. The specimens kept on the list are shown in green, while the specimens removed are shown in orange. The specimens that were not determined to species, but were later included by a taxonomist are shown in purple.

The list of plant species collected in the PEFG was based on data obtained from four databases: JABOT GERAL (Jardim Botânico do Rio de Janeiro, http://jabot.jbrj.gov.br/v3/consulta.php), JABOT RB (http://rb.jbrj.gov.br/v2/consulta.php), REFLORA (Herbário Virtual Reflora, http://reflora.jbrj.gov.br) and speciesLink (INCT Herbário Virtual da Flora e dos Fungos, http://inct.splink.org.br). The databases were accessed on 10 May 2022 and the records were filtered using the following criteria = “Forno Grande”. Our searches returned a total of 16,288 specimens (JABOT GERAL = 6,201; JABOT RB = 1,891; REFLORA = 3,133; speciesLink = 5,063; Suppl. material 1). We manually selected all specimens identified at species level, which led to: JABOT GERAL determined = 4,757, undetermined = 1,444; JABOT RB determined = 1,651, undetermined = 273; REFLORA determined = 2,430, undetermined = 703; and speciesLink determined = 3,801, undetermined = 1,351; Fig. 1).

We then removed duplicates based on name, collector number and year of collection and selected one record per species, prioritising those records with digitised specimens. We also excluded records whose locations do not fall within the coverage area of the PEFG. Finally, we updated the species names according to Flora and Funga of Brasil (http://floradobrasil.jbrj.gov.br) using Plantminer (Carvalho et al. 2010). This online tool accesses information systematised in this web catalogue and verifies taxonomic information on plant species. After these corrections, the preliminary list, with 955 species, was sent to plant taxonomists to check and validate determinations using images in online databases. Intraspecific taxonomic categories and hybrids were not considered. The checklist of the vascular plants of the PEFG was published by Bochorny et al. (2022) and is available in the “Catálogo de Plantas das Unidades de Conservação do Brasil” (https://catalogo-ucs-brasil.jbrj.gov.br/descr_areas.php?area=FornoGrande).

**Origin, endemism and conservation status**

We verified the information regarding the origin of species (native or non-native) following the Flora and Funga of Brazil website (http://floradobrasil.jbrj.gov.br). Additionally, we classified species as endemic to the Atlantic Forest when their geographic distribution is restricted to this domain. The conservation status of the species follows the CNCFlora/JBRJ database (Official National Red List published by MMA Ordinance No 148/2022; MMA (2022)), which serves as the IUCN SSC Brazil Plant Red List Authority (IUCN SSC BP-RLA).

## Geographic coverage

### Description

**Figure 2.**
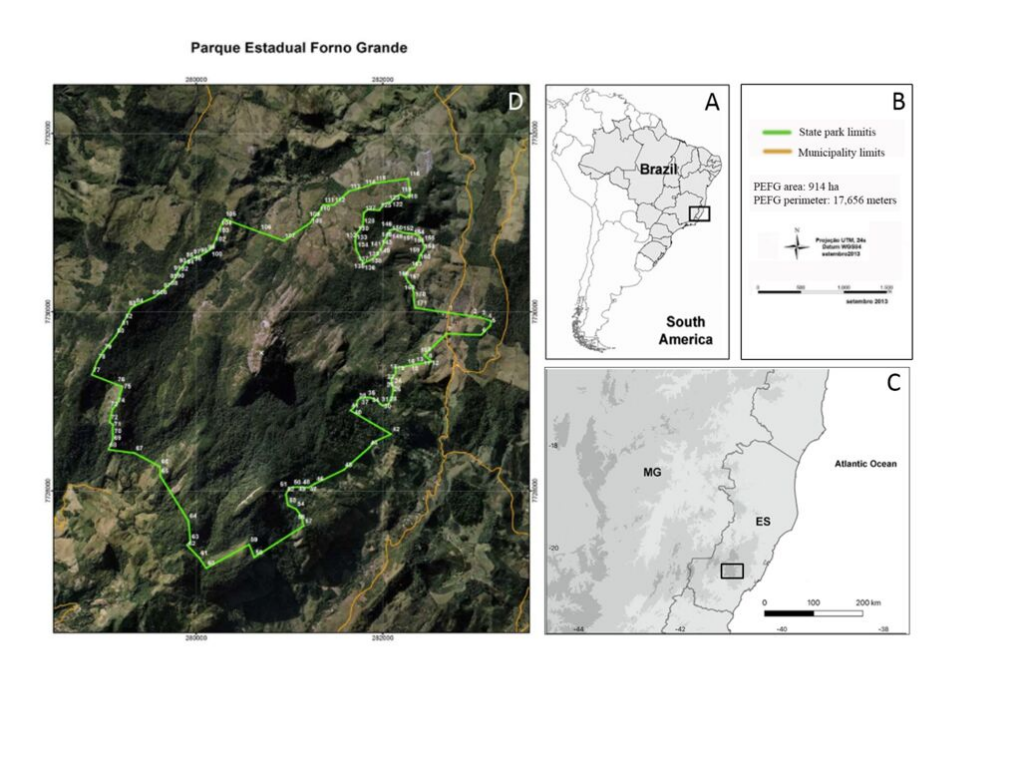
Map of the Parque Estadual do Forno Grande (PEFG). **A** Location of Espírito Santo State in Brazil; **B** PEFG area and perimeter; **C** Location of PEFG in the Espírito Santo State; **D** PEFG limits (perimeter coordinates represented by their record numbers, map adapted from IEMA 2013).

**Figure 3.**
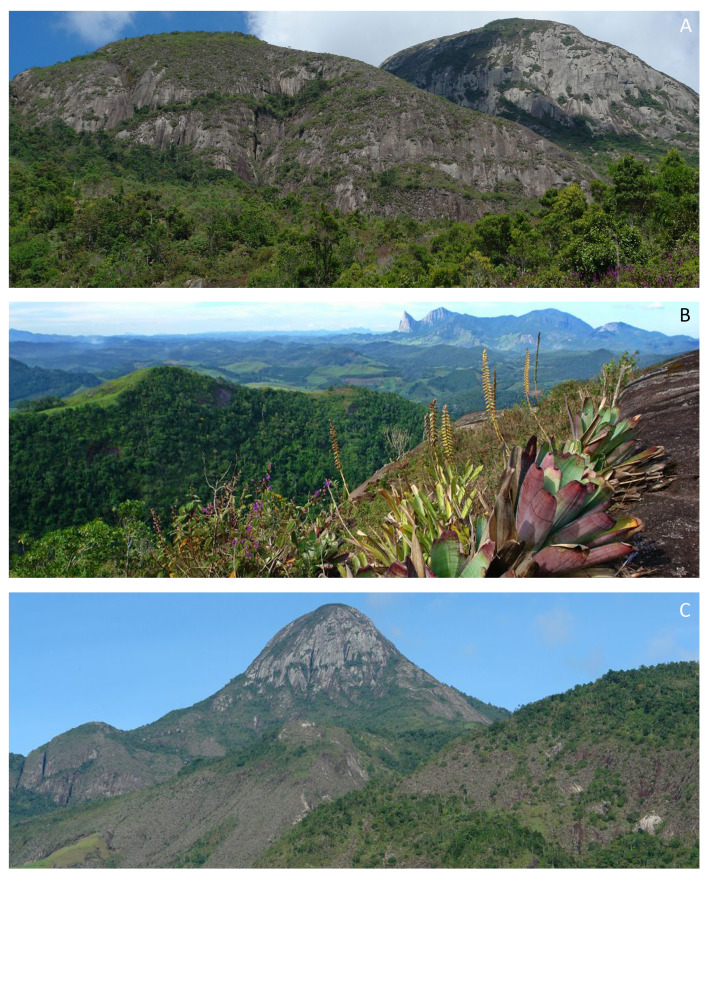
Landscapes and vegetation types of the Parque Estadual do Forno Grande, Espírito Santo, Brazil. **A** Dense montane and high-montane ombrophilous forests; **B** Rocky outcrops amongst the high-altitude forests characterised by predominantly herbaceous/shrubby rupicolous vegetation; **C** “Pico do Forno Grande,” a large granite massif and the second-highest point in Espírito Santo (Photos: Claudio N. Fraga).

The PEFG is a fully Protected Area inserted in a mountainous region considered by some authors as part of Serra do Mar (Scaramuzza et al. 2011) and by others as Serra da Mantiqueira (Silva et al. 2018). It is located in the Municipality of Castelo, in the southern part of the State of Espírito Santo, at the coordinates 20°31'13.74"S and 41°6'21.56"W (Fig. 2), being part of the Mantiqueira Mosaic of Protected Areas (Peixoto and Vargas 2024). Established in October 1960 as a Forest Reserve, it became a state park through Decree No. 7.528 in 1998. The park covers an area of approximately 914 hectares, with altitudes ranging from 1,128 to 2,039 metres, the highest point being the “Pico do Forno Grande,” a large granite massif and the second-highest point in Espírito Santo (IEMA 2000; IEMA 2013). The park's vegetation consists of dense montane to high-montane ombrophilous and seasonal semi-deciduous forests and also rocky outcrops (inselbergs) covered with predominantly herbaceous and shrubby rupicolous vegetation (Fig. 3).

## Taxonomic coverage

### Description

**Figure 4.**
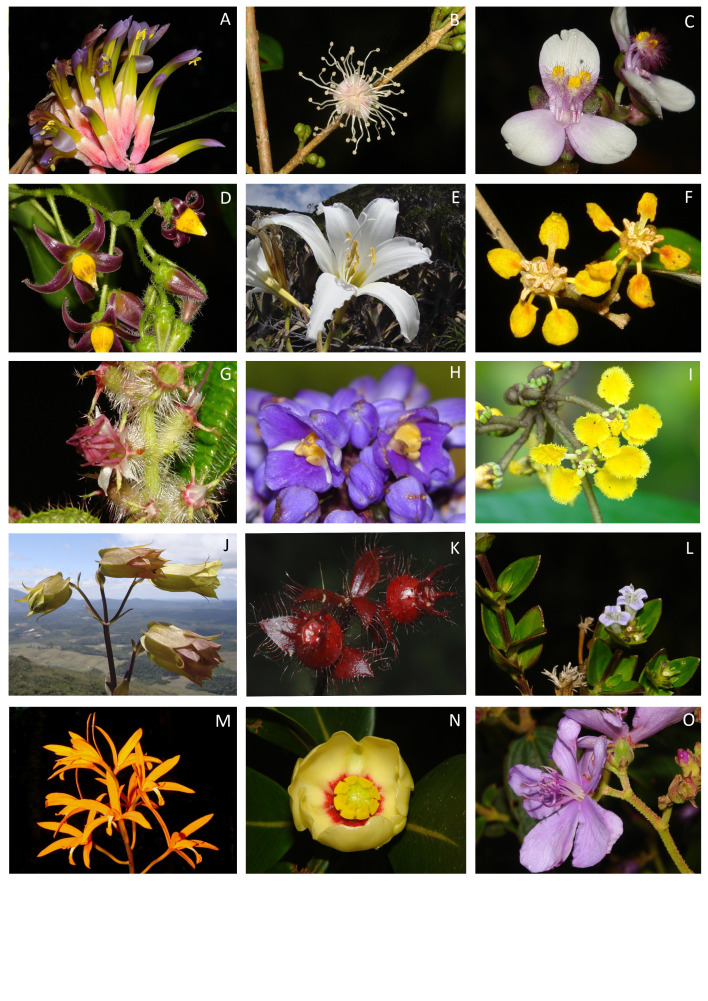
Angiosperms of Parque Estadual do Forno Grande, Espírito Santo, Brazil. **A** *Billbergiaeuphemiae* E.Morren; **B** *Eugeniarepanda* O.Berg; **C** *Tripogandradiuretica* (Mart.) Handlos; **D** *Solanumluridifuscescens* Bitter; **E** *Hippeastrumbrasilianum* (Traub & J.L.Doran) Dutilh; **F** *Tetrapterysmucronata* Cav.; **G** *Leandramultiplinervis* (Naudin) Cogn.; **H** *Dichorisandraprocera* Mart. ex Schult. F.; **I** *Stigmaphyllonalternifolium* A.Juss.; **J** *Prepusaviridiflora* Brade; **K.** *Pleiochitonblepharodes* (DC.) Reginato et al; **L** *Bradeamontana* Brade; **M** *Cattleyacinnabarina* (Bateman ex Lindl.) van den Berg; **N** *Clusiamexiae* P.F.Stevens; **O** *Pleromacastellense* (Brade) P.J.F.Guim. & Michelang (Photos: A, B, C, D, F, G, H, I, L, M, N, O: Claudio N. Fraga; E, J: Paulo Labiak; K: Renato Goldenberg).

**Figure 5.**
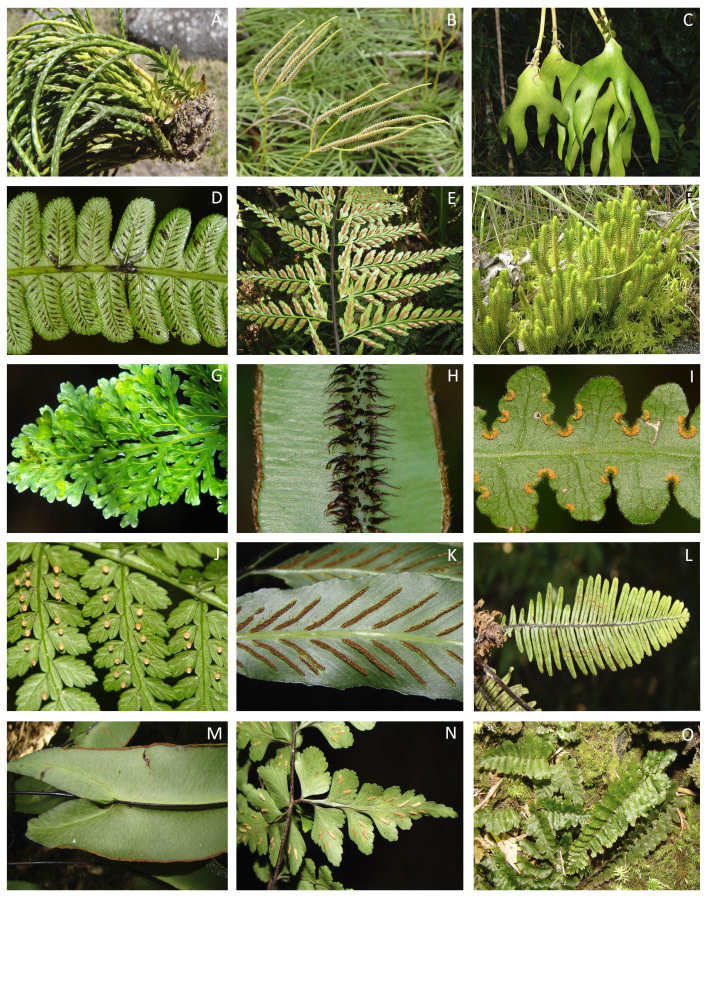
Ferns and lycophytes of Parque Estadual do Forno Grande, Espírito Santo, Brazil. **A** *Phlegmariurushexastichus* (B.Øllg. & P.G.Windisch) B.Øllg.; **B** *Diphasiastrumthyoides* Willd.; **C** *Cheiroglossapalmata* (L.) C.Presl; **D** *Diplaziumlindbergii* (Mett.) Christ; **E** *Aspleniumgastonis* Fée; **F** *Phlegmariurusreflexus* (Lam.) Trevis.; **G** *Hymenophyllumcaudiculatum* Mart.; **H** *Elaphoglossumprestonii* (Baker) J.Sm.; **I** *Blotiellalindeniana* (Hook.) R.M.Tryon; **J** *Eupodiumkaulfussii* (J.Sm.) J.Sm.; **K** *Aspleniumoligophyllum* Kaulf.; **L** *Peclumafilicula* (Kaulf.) M.G.Price; **M** *Doryopterissagittifolia* (Raddi) J.Sm.; **N** *Aspleniumpseudonitidum* Raddi; **O** *Trichomanespilosum* Raddi (Photos: A, B, C, E, F, K, L, M, N, O: Paulo Labiak; G, H, I, J: Claudio N. Fraga).

**Figure 6.**
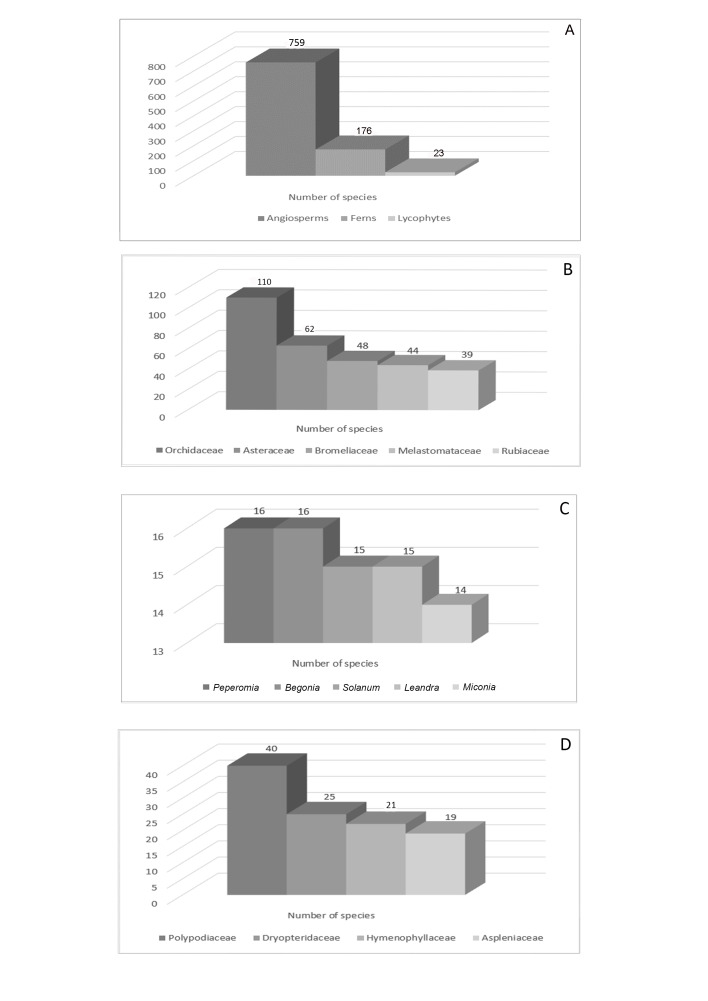
Number of species by plant groups in the Parque Estadual do Forno Grande, Espírito Santo, Brazil., **A.** Angiosperms, ferns and lycophytes; **B.** Richest families; **C.** Richest genera of angiosperms; **D.** Richest families of ferns in Parque Estadual do Forno Grande, Espírito Santo, Brazil.

The plant list of the PEFG includes a total of 958 species (Suppl. material 1) in 414 genera and 115 botanical families, of which 759 are angiosperms (343 genera and 97 families; Fig. 4 and Fig. 6a), 176 are ferns and 23 are lycophytes (71 genera and 18 families; Fig. 5 and Fig. 6a).

The richest angiosperm families in PEFG are Orchidaceae (110 species), Asteraceae (62), Bromeliaceae (48), Melastomataceae (44) and Rubiaceae (39) (Fig. 6b). These families represent 31.3% (303 species) of the total species found in the PEFG. All of these families are also included in Atlantic Forest's top ten richest angiosperm families (BFG Brazilian Flora Group 2022) and identified as the richest in the Espírito Santo State and Brazil (Flora e Funga do Brasil 2024). The richest angiosperm genera are *Peperomia* (16 species), *Begonia* (16), *Solanum* (15), *Leandra* (15) and *Miconia* (14) (Fig. 6c) representing 6.2% of the total species. As for ferns, the richest families are Polypodiaceae (40), Dryopteridaceae (25), Hymenophyllaceae (21), Aspleniaceae (19) and Pteridaceae (18) (Fig. 6d). The richest genera in ferns are *Asplenium* (18), *Elaphoglossum* (15), *Pleopeltis*, *Phlegmariurus* and *Hymenophyllum* (nine species each) and *Phlegmariurus* (9) and *Sellaginela* (8) in lycophytes.

Considering the park size, the number of records, species richness and species cited as threatened for this protected area is considerably high, even for the Atlantic Forest standards. The PEFG presents about one species per hectare (a total of 958 species in 914 hectares). This is notable compared to those recorded in Parque Nacional do Itatiaia (a total of 2,316 species in 30,000 hectares, with 1,280 endemics to the Atlantic Forest and 81 cited as threatened at national level; Moreira (2020a)) and Parque Nacional do Caparaó (a total of 1,791 species in 31,853.12 hectares, with 891 endemics and 63 cited as threatened; Moreira (2020b)).

## Traits coverage

**Origin, endemism, and conservation status**

The PEFG vascular plant list includes 947 native species and 11 non-natives for Brazil. We found 423 endemic species of the Atlantic Forest, of which 344 are angiosperms, 69 are ferns and 10 are lycophytes. The families with the highest number of species that are endemic to the Atlantic Forest are Orchidaceae (59 species), Bromeliaceae (36), Melastomataceae (25), followed by Polypodiaceae (23), Rubiaceae (18) and Piperaceae (17).

The PEFG is home to 951 species that have already been evaluated regarding their conservation status in Brazil. There are 58 threatened species, of which six are considered Critically Endangered (CR), 39 Endangered (EN) and 13 Vulnerable (VU) (Table 1, Suppl. material 1). Amongst the threatened species, 51 are endemic to the Atlantic Forest. Additionally, the park hosts seven species categorised as Data Deficient (DD), 12 species accessed as Near Threatened (NT), 628 species as Not Evaluated (NE) and 256 as Least Concern (LC).

## Temporal coverage

### Notes

The botanical collections by A. C. Brade, dating back to the first half of the 20^th^ century and gathered at the RB Herbarium, are particularly noteworthy. Similarly, the collections by L. Kollmann (2000 to 2008) stored in the MBML Herbarium deserve special mention. Furthermore, collections made between 2006 and 2008 for the project entitled "Diversity of Vascular Flora and Conservation Status of Endemic Species in Three Conservation Units of the Atlantic Forest in the State of Espírito Santo," led by A.M.A. Amorim, A.P. Fontana, R.C. Forzza, C.N. Fraga, L.J.C. Kollmann, P.H. Labiak and R. Goldenberg, are stored in the CEPEC, MBML, RB and UPCB Herbaria. These collections have significantly expanded our understanding of the PEFG flora.

## Usage licence

### Usage licence

Creative Commons Public Domain Waiver (CC-Zero)

## Data resources

### Data package title

Vascular plant list of Parque Estadual do Forno Grande, Brazil

### Number of data sets

1

### Data set 1.

#### Data set name

Database_PE_FornoGrande_2.1

#### Data format

CSV (UTF-8)

#### Description

Our database contains a list of 958 vascular plant species in the PEFG, including information on taxonomic names, herbarium vouchers, source database, collector's name and number, origin, conservation status and endemism in the Brazilian Atlantic Forest.

**Data set 1.** 

Column label	Column description
ProtectedArea	Name of the Brazilian Protected Area.
PlantGroup	Plant Group (Angiosperms or Ferns and Lycophytes).
Family	Plant family.
Genus	Plant genera.
Species	Epithet of the species.
Author	Name of the species author.
taxonID	Family plant , species name and author.
Barcode	Herbarium voucher.
Database	Source database.
Herbaria	Acronym for each herbarium.
CollectorName	Collector's name.
CollectorNumber	Collector´s number.
Origin	native or non-native in Brazil.
ConservationStatus	Conservation status according to IUCN and CNCFlora.
EndemismAF	Species Endemic or not in the Atlantic Forest.

## Additional information

The list of vascular plants of the Parque Estadual do Forno Grande indicates that current inventories of the flora of Brazil's Protected Areas improve the political perception of the gaps in the knowledge of the Brazilian flora and subsidise the proposal of efficient conservation actions. Due to the expeditions in the PEFG area, the number of records and the species richness for a protected area are high by the standards expected for the Atlantic Forest.

## Supplementary Material

B5E335CB-B59D-5591-B064-DD4023397C1D 10.3897/BDJ.12.e133976.suppl1 Supplementary material 1 Vascular plant list of Parque Estadual do Forno Grande, Espírito Santo, Brazil Data type occurrences, taxonomic names, endemism, collection codes and conservation status Brief description List of recorded species in Parque Estadual do Forno Grande, Espírito Santo, Brazil including information on taxonomic names, endemism, collection codes and conservation status. File: oo_1160447.csv https://binary.pensoft.net/file/1160447 Bochorny T, Antar GM, Azevedo IHF, Bianchi Junior F, Carrijo TT, Dutra VF, Fontana AP, Fraga CN, Gerace S, Giacomin LL, Gil ASB, Goldenberg R, Gonzaga DR, Heiden G, Koch I, Kollmann LJC, Labiak PH, Lima DF, Marcusso GM, Moraes PLR, Torres-Leite F, Viana PL & Forzza RC

## Figures and Tables

**Table 1.** List of threatened and data deficient species in Parque Estadual do Forno Grande, Espírito Santo State, Brazil, according to CNCFLora/JBRJ database. (EN = Endangered, CR = Critically Endangered, VU = Vulnerable and DD = Data Deficient). * Species endemic to the Atlantic Forest.

**Family**	**Species**	**Category**
Acanthaceae	*Justiciaclausseniana* (Nees) Profice*	EN
Acanthaceae	*Staurogyneveronicifolia* (Nees) Kuntze*	EN
Amaryllidaceae	*Hippeastrumbrasilianum* (Traub & J.L.Doran) Dutilh*	EN
Apocynaceae	*Rauvolfiacapixabae* I.Koch & Kin.-Gouv.*	EN
Araliaceae	*Dendropanaxamorimii* Fiaschi*	EN
Arecaceae	*Syagrusweddelliana* (H.Wendl.) Becc*	EN
Asteraceae	*Austrocritoniarosea* (Gardner) R.M.King & H.Rob.*	EN
Asteraceae	*Cololobusrupestris* (Gardner) H.Rob.*	EN
Asteraceae	*Seneciograciellae* Cabrera*	EN
Asteraceae	*Vernonanthuraalmedae* (H.Rob.) H.Rob.	EN
Begoniaceae	*Begoniaalbidula* Brade*	EN
Begoniaceae	*Begoniacurtii* L.B.Sm. & B.G.Schub.*	VU
Begoniaceae	*Begoniaespiritosantensis* E.L.Jacques & Mamede*	EN
Begoniaceae	*Begoniaitaguassuensis* Brade*	EN
Bignoniaceae	*Lundiadamazioi* C.DC.	VU
Boraginaceae	*Cordiaochnacea* DC.*	EN
Bromeliaceae	*Aechmeaazurea* L.B.Sm.*	VU
Bromeliaceae	*Aechmeatriangularis* L.B.Sm.*	EN
Bromeliaceae	*Alcantareabenzingii* Leme*	CR
Bromeliaceae	*Canistropsisalbiflora* (L.B.Sm.) H.Luther & Leme*	VU
Bromeliaceae	*Canistrumtriangulare* L.B.Sm. & Reitz*	EN
Bromeliaceae	*Nidulariumkautskyanum* Leme*	EN
Bromeliaceae	*Pitcairniadecidua* L.B.Sm.	EN
Bromeliaceae	*Quesneliakautskyi* C.M.Vieira*	VU
Bromeliaceae	*Vrieseafosteriana* L.B.Sm.*	DD
Bromeliaceae	*Vrieseapereirae* L.B.Sm.*	DD
Cactaceae	*Hatioracylindrica* Britton & Rose*	DD
Cactaceae	*Rhipsalishoelleri* Barthlott & N.P.Taylor*	EN
Cactaceae	*Schlumbergerakautskyi* (Horobin & McMillan) N.P.Taylor*	EN
Chrysobalanaceae	*Licaniaindurata* Pilg.*	EN
Dicksoniaceae	*Dicksoniasellowiana* Hook.	EN
Dryopteridaceae	*Elaphoglossumacrocarpum* (Mart.) T.Moore*	VU
Ericaceae	*Gaylussaciacaparoensis* Sleumer*	EN
Eriocaulaceae	*Paepalanthusmacaheensis* Körn.*	EN
Fabaceae	*Mantiqueirabella* (Mart. ex Benth.) L.P.Queiroz*	DD
Gentianaceae	*Prepusaviridiflora* Brade*	EN
Lentibulariaceae	*Genlisealobata* Fromm*	EN
Lycopodiaceae	*Phlegmariurusnudus* (Nessel) B.Øllg.*	EN
Malpighiaceae	*Byrsonimavernicosa* Nied.*	VU
Malvaceae	*Callianthesellowiana* (Klotzsch) Donnel*	EN
Melastomataceae	*Pleromacastellense* (Brade) P.J.F.Guim. & Michelang.*	CR
Moraceae	*Coussapoapachyphylla* Akkermans & C.C.Berg*	EN
Myrtaceae	*Eugeniacinerascens* Gardner*	CR
Myrtaceae	*Eugeniagoiapabana* Sobral & Mazine*	EN
Myrtaceae	*Siphoneugenadelicata* Sobral & Proença*	VU
Myrtaceae	*Siphoneugenakuhlmannii* Mattos*	VU
Orchidaceae	*Cattleyakautskyana* (V.P.Castro & Chiron) van den Berg*	CR
Orchidaceae	*Cattleyapygmaea* (Pabst) van den Berg	EN
Orchidaceae	*Cattleyawittigiana* (Barb.Rodr.) van den Berg	EN
Orchidaceae	*Grandiphyllumdivaricatum* (Lindl.) Docha Neto*	VU
Orchidaceae	*Octomeriachamaeleptotes* Rchb.f.*	VU
Orchidaceae	*Pabstiellacurti-bradei* (Schltr.) Luer*	VU
Orchidaceae	*Polystachyarupicola* Brade*	CR
Orchidaceae	*Zygopetalumjugosum* (Lindl.) Schltr.*	EN
Poaceae	*Merostachysburmanii* Send.*	EN
Polypodiaceae	*Mycopterissemihirsuta* (Klotzsch) Sundue*	EN
Polypodiaceae	*Pleopeltisalborufula* (Brade) Salino*	EN
Pteridaceae	*Doryopterisrediviva* Fée*	VU
Pteridaceae	*Jamesoniabiardii* (Fée) Christenh.*	EN
Rubiaceae	*Bradeaanomala* Brade*	EN
Rubiaceae	*Bradeamontana* Brade*	CR
Rubiaceae	*Palicoureaforsteronioides* (Müll.Arg.) C.M.Taylor*	DD
Rubiaceae	*Palicoureamalaneoides* (Müll.Arg.) C.M.Taylor*	DD
Urticaceae	*Coussapoapachyphylla* Akkermans & C.C.Berg*	EN
Urticaceae	*Pilearhizobola* Miq.	DD
